# The impacts of quality improvement on maternal and newborn health: preliminary findings from a health system integrated intervention in four Ethiopian regions

**DOI:** 10.1186/s12913-020-05391-3

**Published:** 2020-06-08

**Authors:** Ashley K. Hagaman, Kavita Singh, Mehiret Abate, Haregeweyni Alemu, Abera Biadgo Kefale, Befikadu Bitewulign, Abiy Seifu Estifanos, Abiyou Kiflie, Zewdie Mulissa, Hillina Tiyo, Yakob Seman, Meseret Zelalem Tadesse, Hema Magge

**Affiliations:** 1 Department of Social and Behavioral Sciences, Yale School of Public Health, Yale University, 60 College St, New Haven, CT 06510 USA; 2 Carolina Population Center, University of North Carolina at Chapel Hill, 123 W. Franklin St, Chapel Hill, NC 27516 USA; 3 Yale School of Public Health, 135 College St, New Haven, CT 06510 USA; 4 Department of Maternal and Child Health, Gillings School of Global Public Health, University of North Carolina at Chapel Hill, 135 Dauer Dr, Chapel Hill, NC 27599 USA; 5 Institute for Healthcare Improvement, Addis Ababa, Ethiopia, Addis Ababa, Ethiopia; 6 Department of Reproductive Health, School of Public Health, Addis Ababa University, Zambia Street, Tikur Anbessa Hospital Building, Lideta Sub-city, Addis Ababa, Ethiopia; 7 Federal Ministry of Health, Ethiopia, Sudan Street, Addis Ababa, Ethiopia; 8 Division of Global Health Equity, Brigham and Women’s Hospital, 75 Francis St, Boston, MA 02115 USA

**Keywords:** Maternal health services, Quality improvement, Neonatal health services, Healthcare delivery

## Abstract

**Background:**

Quality improvement (QI) methods are effective in improving healthcare delivery using sustainable, collaborative, and cost-effective approaches. Systems-integrated interventions offer promise in terms of producing sustainable impacts on service quality and coverage, but can also improve important data quality and information systems at scale.

**Methods:**

This study assesses the preliminary impacts of a first phase, quasi-experimental, QI health systems intervention on maternal and neonatal health outcomes in four pilot districts in Ethiopia. The intervention identified, trained, and coached QI teams to develop and test change ideas to improve service delivery. We use an interrupted time-series approach to evaluate intervention effects over 32-months. Facility-level outcome indicators included: proportion of mothers receiving four antenatal care visits, skilled delivery, syphilis testing, early postnatal care, proportion of low birth weight infants, and measures of quality delivery of childbirth services.

**Results:**

Following the QI health systems intervention, we found a significant increase in the rate of syphilis testing (ß = 2.41, 95% CI = 0.09,4.73). There were also large positive impacts on health worker adherence to safe child birth practices just after birth (ß = 8.22, 95% CI = 5.15, 11.29). However, there were limited detectable impacts on other facility-usage indicators. Findings indicate early promise of systems-integrated QI on the delivery of maternal health services, and increased some service coverage.

**Conclusions:**

This study preliminarily demonstrates the feasibility of complex, low-cost, health-worker driven improvement interventions that can be adapted in similar settings around the world, though extended follow up time may be required to detect impacts on service coverage. Policy makers and health system workers should carefully consider what these findings mean for scaling QI approaches in Ethiopia and other similar settings.

## Background

Global improvements in maternal and neonatal mortality and morbidity are laudable, attributable to interventions improving quality and access to care across the antenatal, delivery, and postnatal periods [1–3]. However, disparities persist, particularly in rural areas and within marginalized populations with limited access to education and social mobility [4]. Increasing coverage of maternal and newborn health interventions relies on improving service quality and demand for services. Quality improvement (QI) methods are intended to enhance the delivery of effective health interventions using sustainable, collaborative, and cost-effective approaches. Quality is also considered a key element in efforts to better health outcomes and improve service delivery [5–8]. QI interventions that include health care provider training and mentorship may be highly effective for improving both patient outcomes and provider performance [9, 10]. QI interventions have demonstrated some success in improving some maternal and child health outcomes in low-income countries [11–13]. For example, in Ghana, a systems-integrated continuous QI intervention deployed through learning collaboratives and quality improvement teams improved skilled delivery and antenatal care coverage and reduced under-five mortality [12]. A similar QI model deployed in Tanzania and Uganda increased the receipt one of four evidence-based essential interventions for maternal and newborn care [13]. In rural Rwanda, QI strategies including learning collaboratives and mentoring were determined feasible and promising for improving neonatal outcomes [14].

While these studies indicate promise, they also demonstrate a need for further refinement and testing of these strategies, particularly in low-income contexts [15]. A systematic review reported that implementing multiple quality improvement strategies, such as combined training and supervision, targeted at multiple community and system levels, may be particularly effective in low-income settings [16]. Such systems-integrated interventions require enormous coordination and commitment, and models that demonstrated feasibility and efficacy may offer important contributions to further improvements in maternal and newborn mortality and morbidity in resource constrained contexts [6].

In Ethiopia, the 2019 Demographic and Health Survey indicates that 74% of women received antenatal care from a skilled provider and 43% received four or more ANC visits [17]. Additionally, less than 48% of women delivered at a health facility. Additionally, despite great achievements in reducing maternal and neonatal deaths in the last two decades, Ethiopia continues to have high maternal mortality with 412 maternal deaths per 100,000 live births and neonatal mortality with 29 neonatal deaths per 1000 live births [18]. While maternal health coverage has greatly improved over time, these findings indicate persistent disparities and unmet demand and need for quality healthcare [17]. Ethiopia’s government has prioritized quality and equity as one of four pillars of the Health Sector Transformation Plan (HSTP). QI methods may be one important strategy to achieve these goals. The Institute for Healthcare Improvement (IHI) is supporting the Federal Ministry of Health in Ethiopia (FMoH) in implementing a comprehensive QI initiative focusing on quality planning, building capability in quality management and improvement at all health system levels, and designing and testing a scalable district-wide approach for health improvement with a focus on maternal and newborn health. University of North Carolina at Chapel Hill and Addis Ababa University serve as the external evaluation partners to this initiative.

In this study, we evaluate whether the QI initiative is leading to changes in key maternal and newborn health outcomes during its pilot phase in 4 district-based improvement collaboratives across 4 regions of Ethiopia: Oromia, Tigray, Amhara, and Southern Nations, Nationalities, and Peoples’ Region (SNNPR). A key element of the district QI initiative is the training of QI teams to implement locally-derived, systems-embedded, change ideas or “interventions”. These interventions are intended to improve service delivery and create demand for services. Project staff worked alongside district coaching teams to visit facilities to coach and mentor teams as they develop and test change ideas. In this paper we employed an interrupted multivariable time series analysis to understand if, and how, the intervention is leading to improved maternal and newborn health outcomes during the initial phase. We also assessed if particular intervention components were associated with impact, which could subsequently be targeted when bringing the intervention to scale at the national level.

## Methods

### QI initiative and program implementation

**Fig. 1:**
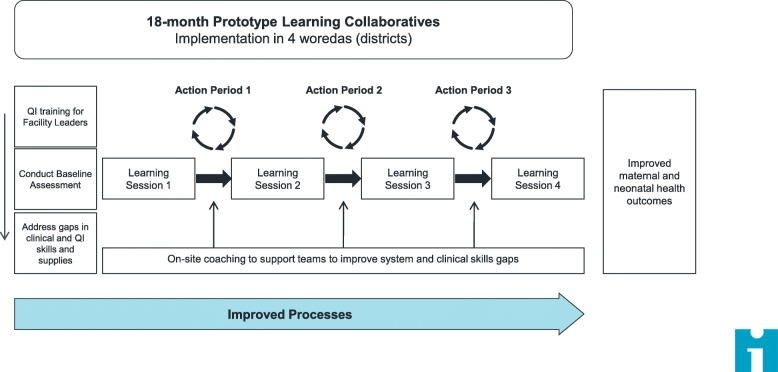
The Ethiopia Quality Improvement Intervention Components

In partnership with the Federal Ministry of Health, the program established governmental district-wide learning collaboratives and provided them with structured, systematic, QI and relevant clinical skills training. The pilot phase implemented four learning collaboratives (one in each primary hospital catchment area that included all government health facilities (health centers and their corresponding health posts) in its referral network), beginning in September 2016 and ending in September 2018. Each collaborative formed quality improvement teams (QITs) that work with support from government leadership at multiple levels (e.g., woreda, zonal, regional) within each site. Each health center QIT included health extension workers (HEWs) from its linked health posts. Primary hospitals are the first point of contact with physicians and provide care for complications including caesarian section, and blood transfusions. Health centers are nurse/health officer-led and provide primary health care services, including uncomplicated deliveries. Health posts are managed by a health extension worker and provide basic health services at the lowest administrative level. QIT participants included facility heads, maternal and neonatal clinical staff, data officers, and health extension workers. The pilot phase was implemented in one collaborative in 4 agrarian and 1 pastoralist (data collection ongoing) region to represent diversity in the country. These data are from the first four completed collaboratives, including 30 QITs. QITs attended four structured learning sessions over 18 months for training in QI, experience sharing, and peer learning, followed by the implementation of team-initiated QI ‘change ideas’ and troubleshooting. In between the learning sessions, intensive coaching visits were made by project staff to supervise and mentor the QITs (see Fig. 1 for intervention components). The results presented in this paper use data from the pilot phase, including between 9 and 13 months of pre-intervention data per facility, and follow outcomes until December 2018, totaling 878 facility months across all pilot facilities. The QI intervention was considered to reach full implementation between the 2nd and 3rd learning session, the time at which change ideas were developed, tested, and monitored. Additionally, staff knowledge acquisition of QI methods and strategies would not be sufficient to conduct the aforementioned interventions until this timepoint.

### Intervention

We consider the intervention as having three ‘active ingredients’, including the clinical and QI trainings done at the collaborative start, the change ideas tested by the QITs, and coaching visits provided to support clinical quality and coach QITs. We characterize the main ingredient of the intervention using the change ideas tested within each QIT. QITs developed change ideas targeting these maternal and child health indicators. Multiple change ideas targeting one or more priority areas were defined and tested within each QIT team at their respective facilities. Information for each change idea was systematically documented into a project monitoring database and included the date initiated, the implementation strategy, and specific goals, targets, and timelines as part of QI coaching. This change idea data was extracted and dichotomized so that, if any change was developed and tested for a particular target indicator, the facility was coded as having tested a change in that month. We also created an overall category of any change tested in any category over the intervention period as well as a continuous count of the total changes tested across all categories. Coaching included observing clinical care and supporting health care workers’ clinical skills, motivating QITs, supporting facility leadership in fostering team communication and identifying problems, and supporting developing and monitoring testable solutions to address gaps in care. Daylong coaching visits were made to QITs one to two times per month over the course of the support phase (between 11 and 15 months). Additionally, in order to simultaneously ensure outcome data quality and strengthen the routine data reporting system (the government health management information system (HMIS)), all facility data were extracted from the facility paper registers. This data was validated by comparing and reconciling with HMIS reports as a part of data quality improvement efforts.

### Outcomes

Outcome data were extracted from facility registers from May 2016 until at least 6 months following the fourth (and last) learning session. In this paper, we present results of the intervention on the following maternal and newborn health outcomes: skilled delivery (the proportion of births attended by a skilled birth attendant); four antenatal care visits (ANC) completed (the proportion of pregnant women who have four ANC visits by 36 weeks of pregnancy); syphilis testing (proportion of ANC users who have been tested for syphilis); neonatal complications (proportion of cases treated for sepsis and asphyxia); early PNC (the proportion of women who receive postnatal care (PNC) within 48 h of delivery); and proportion of low birth weight infants. Infants placed in Kangaroo Mother Care were also examined as an outcome, but this data is only captured at the hospital level, and thus are not included in the longitudinal analyses. Outcome variables were calculated using census-derived population estimates to calculate the denominators of number of expected pregnancies and number of expected live births as per the definitions in HMIS (Commission; 2008).

We also explore the extent to which the QI intervention improved compliance to three bundles of essential birth practices for safe childbirth. These included: On-Admission Safety Bundle; Before Pushing Safety Bundle, and Just After Birth Safety Bundle). Bundle components can be found in supplementary Table S.2. These bundles outline essential components to the standard of routine maternal care, and were derived from the WHO Safe Childbirth Checklist which had been adopted by the Ethiopia Federal Ministry of Health and introduced to health care facilities at the time of program initiation [19]. The Checklist was introduced as a job aid for clinical care provision as part of the QI initiative in the first learning session, and implemented in line with similar studies in LMIC [20]. Bundle adherence was assessed through the triangulation of three methods. First, monthly retrospective medical record charts of 30 randomly selected births from a facility were reviewed for documentation of bundle elements; second, senior program officers observed all births that occurred during a coaching visit and checked if each element was conducted; and third, each paper copy of the safety birth checklist were assessed for completeness. Adherence was considered achieved (and coded as ‘1’) if all element of the particular bundle were met for a given birth. If any element of the three bundles was not performed, adherence was not achieved (coded as ‘0’). Facilities kept monthly logs of the proportion of births with 100% adherence to a given bundle.

### Facility-level variables

We expect that the type of facility and geography may affect the magnitude, speed, and type of change that is possible following QI changes, particularly given the large amount of facilities included in the intervention. For example, some regions have richer resource chains, more highly skilled or experienced staff, or environments more conducive to change compared with others. To address some of these differences, we control for selected covariates at the facility level, collected from a baseline assessment. These included facility type (health center vs. hospital), its catchment population, the number of staff working within each facility, and the geographic region of each facility. A baseline survey of each health facility assessed the presence or absence of essential pharmacy supplies, medicines, and laboratory testing equipment required to provide minimal acceptable services related to maternal care and child delivery. From each of these identified indicators, we created a ‘medication index’ to reflect this baseline facility quality (see supplementary material) and include this as a covariate in all models.

### Analysis

First, we compared the pre and post-intervention means for our outcomes. The quasi-experimental design of the intervention, whereby each facility serves as its own control over time, allows us to leverage an interrupted time series (ITS) approach [21]. This analytic strategy has been employed in public health intervention evaluations with access to systematic longitudinal data [22]. ITS uses a segmented multivariable regression to detect whether the intervention (e.g., the change ideas tested) is associated with a significant trend shift in the outcome variable of interest (e.g., proportion of women receiving postnatal care within 48 h of delivery, etc.). The ‘interruption’ (e.g., the intervention), was considered present after the project met full implementation so that the pre-trend uses approximately 13 months of data per facility and the post-trend uses about 20 months, allowing us to account for seasonality effects. The core equation to be estimated using GEE was:

$$ {Y}_{ft}=\kern0.5em \alpha +{\beta}_1\bullet t+{\beta}_2\bullet {CT}_f+{\beta}_3\bullet \left({CT}_f\bullet t\right)+{\beta}_4\bullet {X}_f+{\mu}_{ft} $$

*Y* is the outcome of interest, *t* is the time period*, f* is the facility, and *CT*_*f*_ represents pre/post intervention (0 if before full implementation and 1 if after) or a change category tested in facility *f*. The vector *X* represents the facility and covariates included in the model (facility type, catchment population, the geographic woreda, and the baseline medicine index). The immediate impact of the ‘interruption’ is indicated by β_2_ and determines whether there is a one-time jump in the outcome value after full implementation of the intervention or change. β_3_ indicates the longer term impact or trend, indicating whether there is a change in the slope of *Y* after full implementation of the intervention or change (the difference in slope from before to after). We examine both of these effects in order to understand the immediate impact of the intervention as well as if this effect was maintained and sustained in the 15–23 months following the intervention’s full implementation. In addition, β_2 +_ β3 yields the overall effect of the change category when time equals 1_._ We also include a marker of intervention intensity, number of coaching visits, to explore if this ‘ingredient’ had an independent impact, particularly because this dimension of the intervention is adjusted as the program scales nationally. Changes targeting different categories were each modeled in separate multivariable time series regressions. A non-linear trend is accommodated through a quadratic term in *t* multiplied by the change variable and models also include controls for time and time squared (not shown in the equation).

## Results

A description of the intervention facilities and their characteristics is provided in Table 1. Health centers and hospitals serve as the administrative and referral center to an average of 4.1 health posts. QIT facilities employed on average 48 staff (including health workers, technicians, and cleaning staff) on average and were responsible for serving populations of around 35,700. QITs were provided on average about 20 coaching visits over the course of the intervention’s learning sessions, targeting at least one per month.

**Table 1:** Program and facility-level characteristics

Variable	N (***n*** = 30)	% or mean	Range (if applicable)
Health center (versus hospital)	27	90.0	
Health posts per center		4.1	1–10
Mean number of staff		48	16–159
Mean number of coaching visits		19.6	10–34
*QITs per Collaborative*			
Bokoji/Lemmu Bilbilu	8	26.7	
Tanqua Abergele	6	20.0	
Daguna Fango	6	20.0	
Fogera	10	33.3	
Catchment population		35,700	11,600 – 236,600

Table 2 includes examples of change ideas tested within the program and the health indicators that were targeted. QITs tested change ideas targeting the majority of indicators and tested between two and three different ideas within each category. The total number of change ideas tested ranged from six to 21, depending on QIT.

**Table 2:** Change categories, an example of an idea tested within one category, and the number of PHCUs testing each change

Change Category	Examples of change ideas	Expected outcomes impacted	QITs implementing change (*n* = 30)	Mean changes tested (range)^**a**^
ANC	Host a community pregnant women’s forum for experience sharing, help with health care navigation, and education	Syphilis testing; ANC coverage; skilled delivery; PNC; care of low birth weight newborns	29	2.7 (1–5)
Syphilis	Task shifting of syphilis testing from lab technicians to midwives, nurses and health officers, when technicians not available	Syphilis testing	16	2.4 (1–4)
Skilled delivery	Offer visits and tours of delivery rooms to pregnant mothers to develop relationships and engage them in care early	Skilled delivery; PNC; low birth weight	23	2.3 (1–4)
PNC	Text/call community health workers to remind them to visit mother at her home within 48 h of birth	PNC	28	3.0 (1–6)
Total Changes			30	13.8 (6–21)

^**a**^Does not include facilities that did not test a change within the respective category

Unadjusted pre-post intervention comparisons are presented in Table 3. We find significant improvements among many of the target indicators. The mean proportion of mothers attending at least four ANC visits increased from 64.1 to 75.3% (*p* < 0.001). Similarly, facilities increased the testing of mothers attending ANC for syphilis (from 54.7 to 68.5%, *p* < 0.001) and were more likely to ensure mothers received a PNC visit within 48 h of discharge (from 49.4 to 58.2%, *p* < 0.001). Additionally, the proportion of infants who received appropriate treatment in the event of birth asphyxia, sepsis, KMC, and low birth weight also increased, though only significantly for sepsis treatment (*p* = 0.004). Adherence to each clinical bundle also improved following intervention implementation. The mean skilled delivery coverage appeared to decrease across the intervention period (64.0% pre-intervention and 57.5% post, *p* = 0.01). Overall, facilities reported on average 1.5 maternal deaths and 10.9 perinatal mortalities every month. We report the mean number of maternal, neonatal, stillbirth, and perinatal mortalities along with the total number of deaths in the pre and post intervention periods for each category (see Table 3 for details). Though our sample lacks sufficient size to make any robust statistical inference, compared to the pre-intervention period, across the post-intervention period there was a slight decrease in stillbirth and perinatal mortalities and a slight increase in neonatal and maternal mortalities.

**Table 3:** Pre and post intervention and overall means of maternal and child health outcome variables

Outcomes	Pre-intervention			Post-intervention				Overall		
	Facility months	Mean	SD	Facility months	Mean	SD	t-test	Facility months	Mean	SD
**ANC Processes**										
Four ANC visits	371	64.1	36.1	507	75.3	25.7	< 0.001	878	70.6	31.1
Syphilis Testing during ANC	371	54.7	64.8	506	68.5	51.8	< 0.001	877	62.7	58.0
**Delivery Care**										
Skilled delivery^a^	371	64.0	35.9	507	57.5	35.9	0.01	878	60.2	36.9
*Birth Bundles*										
On Admission	197	45.9	40.0	503	81.0	30.1	< 0.001	700	71.1	36.7
Before Pushing	197	43.7	39.2	503	76.8	32.7	< 0.001	701	67.5	37.8
Soon After Birth	197	45.6	42.9	503	59.5	43.4	< 0.001	701	55.6	43.7
**PNC**										
PNC visit within 48 h	371	49.4	37.9	507	58.2	37.4	< 0.001	878	52.0	37.9
**Management of Complications**^**b**^**/Underweight Infants**										
Neonatal Resuscitation	65	93.5	24.3	124	93.0	23.4	0.554	189	93.2	23.6
Neonatal Sepsis	16	87.5	34.2	84	99	9.1	0.004	100	97.2	16.2
KMC (hospitals only)	19	80.1	34.0	47	96.0	19.0	0.009	66	91.5	25.1
Underweight infants	367	16.6	36.1	504	15.3	33.8	0.703	871	15.8	34.8
**Mortality Outcomes**		Mean (raw count)								
Neonatal mortalities on the first day of life	367	1.0 (19)	5.4	504	1.6 (27)	9.5	0.127	871	1.4 (46)	8.0
Neonatal mortalities (within 7 days)	367	1.5 (28)	7.2	504	1.9 (42)	9.9	0.217	871	1.8 (70)	8.9
Fresh Stillbirths	368	12.0 (143)	55.8	504	10.0 (220)	22.6	0.767	872	10.9 (363)	40.1
Perinatal Mortalities^c^	368	12.1 (171)	55.8	504	10.1 (262)	22.6	0.762	872	10.9 (433)	40.1
Maternal mortalities	367	1.2 (11)	12.8	504	1.8 (21)	18.1	0.309	871	1.5 (32)	16.2

^a^ Skilled deliveries / expected pregnancies

^**b**^ The denominator for neonatal resuscitation and KMC (Kangaroo mother care) is the number of cases detected and the mean is the proportion of cases in which standard of care treatment was provided following asphyxia or low birth weight detection. Underweight infants were any infant weighing below 2500 g at birth

^c^ Perinatal mortality calculated as (Neonatal deaths within 7 days + still births)/ (still births + live births)

Table 4 illustrates the adjusted multivariate regressions of each change category tested on maternal health coverage and neonatal health outcomes. Immediately following the full implementation of the intervention, a significant increase in the rate of syphilis testing was detected (ß = 2.41, 95% CI = 0.09,4.73). Additionally, following the testing of an ANC targeted change idea also had a significant impact on syphilis testing coverage (ß = 2.34, 95% CI = 0.08, 4.60). The trend variable was slightly, and significantly, negative for both of these effects, indicating that the increase in syphilis testing slightly diminished over the year following the intervention (linear trend ß = − 0.20, 95% CI = -0.37, − 0.03 and ß = − 0.17, 95% CI = -0.32, − 0.02 respectively). The overall effects of this change category when time equals 1 (β_2+_ β_3_) were positive at 2.21 for the intervention and 1.17 for ANC. Though not significant, the testing of a change idea targeting ANC resulted in a slight increase the proportion of women receiving four ANC visits (ß = 0.60, 95% CI = − 1.10, 1.45). Finally, testing change ideas targeting ANC and SBA resulted in significant decreases in the proportion of low birth weight infants (ß = -0.22, 95% CI = -0.34, − 0.10 and ß = -0.28, 95% CI = -0.44, − 0.13 respectively). The corresponding linear trend for both change idea targets was slightly positive (though close to zero) indicating that the increase was significantly sustained over time. Thus the overall effects (β_2+_ β_3_) were negative.

**Table 4:** Multivariate regression results of selected outcomes: coefficients and standard deviations for the change categories and trend following the intervention

Model Number and Outcome Indicators															
	(1)	(2)	(3)	(4)	(5)	(6)	(7)	(8)	(9)	(10)	(11)	(12)	(13)	(14)	(15)
**Target indicator**	Syphilis Testing			Skilled Delivery			Early Postnatal Care				Receiving 4 ANC Visits		Proportion of LBW		
	ß(CI)	ß(CI)	ß(CI)	ß(CI)	ß(CI)	ß(CI)	ß(CI)	ß(CI)	ß(CI)	ß(CI)	ß(CI)	ß(CI)	ß(CI)	ß(CI)	ß(CI)
Intervention	2.41*			−0.72			−1.42				0.18		−0.22***		
(pre/post)	(0.09–4.73)			(−1.85–0.42)			(−2.84–0.00)				(− 1.10–1.45)		(− 0.34 - -0.1)		
Linear trend	− 0.20*			0.05			0.11*				−0.01		0.02***		
	(−0.37 - -0.03)			(−0.04–0.13)			(0.01–0.21)				(−0.10–0.08)		(0.01–0.02)		
Syphilis change tested		0.20													
		(−2.26–2.66)													
Syphilis change trend		−0.04													
		(−0.20–0.12)													
ANC change tested			2.34*			−0.54		−0.9				0.6		− 0.22***	
			(0.08–4.60)			(−1.60–0.53)		(−2.23–0.47)				(−0.66–1.85)		(− 0.34 - -0.10)	
ANC change trend			−0.17*			0.03		0.06				−0.04		0.01***	
			(−0.32 - -0.02)			(− 0.04–0.10)		(− 0.03–0.15)				(− 0.13–0.04)		(0.01–0.02)	
SBA change tested					−0.39					−0.2					−0.28***
					(−1.61–0.82)					(−1.82–1.43)					(−0.44 - -0.13)
SBA change trend					0.03					0.03					0.02***
					(−0.05–0.11)					(−0.07–0.13)					(0.01–0.02)
PNC change tested									−0.98						
									(−2.44–0.48)						
PNC change trend									0.06						
									(−0.03–0.16)						
Facility Months	877	874	874	878	875	875	878	875	875	875	878	875	871	868	868
Coaching Visits	0.01	0.01	0.01	0	0	0	0	−0.01	−0.01	− 0.01	0	0	0	0	0
Medicine Index	0.05***	0.05***	0.05***	0.02*	0.02*	0.02*	0	0	0	0	0	0.01	0	0	0

95% Confidence interval in parentheses. *** *p* < 0.001, ** *p* < 0.01, * *p* < 0.05

All models adjusted for: catchment population, Woreda, time and time^2^, coaching visits, medicine index, and facility type. *LBW* low birth weight

The non-linear term (change tested*time^2^ is not displayed because of negligible values

Results did not demonstrate any significant impacts on skilled delivery following exposure to changes targeting skilled birth attendance or increasing ANC. Similarly, the number of coaching visits paid to a particular QIT did not impact any of the MCH coverage indicators. We did find, however, that some aspects of infrastructural capacity, captured through indexes of the presence of essential medicines and equipment, had significant effects. We see a consistent positive impact of the medicine supplies, where an increase in one supply item corresponded to a slight increase Syphilis testing and skilled delivery (ß = 0.05, 95% CI = 0.03, 0.07 and ß = 0.02, 95% CI 0.00, − 0.03 respectively). See Table 4 for all results on maternal health coverage and neonatal health outcomes (the quadratic term is not discussed because of its negligible value).

Finally, Table 5 contains findings of the intervention’s impact on QIT adherence to maternal and neonatal clinical bundles. We find large positive impacts on adherence to the soon after birth bundle following full implementation of the intervention (ß = 8.22, 95% CI = 5.15, 11.29). The linear trends indicate a slight significant decrease over time (ß = -0.61, 95% CI = − 0.85, − 0.37). Thus, the overall effect of this change when time equals 1 (β_2+_ β_3_) was 7.61. Additionally, QITs that implemented a change targeted specific to the soon after birth bundle, saw a significant immediate increase in adherence (ß = 2.71, 95% CI = 0.62, 4.79), with a small decrease over time (ß = -0.17, 95% CI = − 0.31, − 0.04). The overall effect (β_2+_ β_3_) was 2.54. QITs that tested any change and had more total changes also resulted in an increase in soon after birth bundle adherence (total change ß = 0.25, 95% CI = 0.12, 0.39), with small significant decrease observed over time (total change linear trend ß = -0.02, 95% CI = − 0.85, − 0.37). The overall effect (β_2+_ β_3_) was 0.23. We also find significant positive impacts of the intervention on adherence to the on-admission bundle, but only for QITs that tested changes targeted directly to this improvement (ß = 2.16, 95% CI = 0.60, 3.71). Neither the type nor quantify of change ideas implemented significantly improved the trend of adherence for the Before Pushing bundle. Overall, the coefficients and CIs are identical between row 1 (intervention as binary exposure) and row 5 (any change tested), because all facilities tested the bundles.

**Table 5:** Intervention impacts on clinical bundle adherence^1^

		Soon After Birth Bundle Adherence		Before Pushing Bundle Adherence						On-Admission Bundle		
	(1)	(2)	(3)	(4)	(5)	(6)	(7)	(8)	(9)	(10)	(11)	(12)
	ß(CI)	ß(CI)	ß(CI)	ß(CI)	ß(CI)	ß(CI)	ß(CI)	ß(CI)	ß(CI)	ß(CI)	ß(CI)	ß(CI)
Intervention (pre/post)	8.22***				−0.19				1.8			
	(5.15–11.29)				(−2.53–2.15)				(− 0.53–4.13)			
Linear trend	−0.61***				0.03				−0.11			
	(−0.85 - -0.37)				(−0.15–0.22)				(−0.30–0.07)			
Soon after birth bundle change tested		2.71*										
		(0.62–4.79)										
Linear trend		−0.17*										
		(−0.31 - -0.04)										
Before Pushing bundle change tested						0.38						
						(−1.12–1.87)						
Linear trend						−0.03						
						(−0.13–0.06)						
On-Admission bundle change tested										2.16**		
										(0.60–3.71)		
Linear trend										−0.15**		
										(−0.26 - -0.04)		
Any Change Tested			8.22***				−0.19				1.8	
			(5.15–11.29)				(−2.53–2.15)				(−0.53–4.13)	
Linear trend			−0.61***				0.03				−0.11	
			(−0.85 - -0.37)				(−0.15–0.22)				(−0.30–0.07)	
Total changes tested				0.25***				0.02				0.1
				(0.12–0.39)				(−0.08–0.13)				(0.00–0.20)
Linear trend				−0.02***				0				−0.01
				(−0.02 - -0.01)				(−0.01–0.01)				(−0.01–0.00)
Facility Months	701	701	701	701	701	701	701	701	700	700	700	700

^1^Adherence is defined as complete all five components of the clinical bundle (see Supplementary material for component details)

All models adjusted for: catchment population, Woreda, time and time^2^, coaching visits, medicine index, and facility type. The non-linear term (change tested*time^2^ is not displayed because of negligible values

95% Confidence interval in parentheses. *** *p* < 0.001, ** *p* < 0.01, * *p* < 0.05

## Discussion

As low- and middle-income countries continue to strengthen the capacity of their health systems, quality improvement methods are often chosen as a core implementation strategy to meet their goals [23, 24]. This evaluation of the pilot phase of a multipronged quality improvement program identified significant impacts on the quality of care provided over time. For example, changes targeting clinical practices immediately after birth, significantly improved the quality of newborn assessment. Quality improvement changes also significantly improved syphilis testing in antenatal care, and these increases in coverage dropped only slightly over time. Detecting and treating syphilis during pregnancy significantly reduces the likelihood of stillbirth, neonatal death, bone deformities, and cognitive impairment [25]. ANC interventions likely increased syphilis testing coverage because they aim to recruit more women into antenatal care at the health facilities and syphilis testing is performed in their first visit. The intervention as a whole, including testing any change idea, also improved quality of care for delivery, as seen through improved adherence to clinical bundles. Over time, we found only slight decreases in adherence, indicating the feasibility of sustainment of safety procedures in facility settings. Semrau et al. found similar increases in staff adherence to safe birth practices, but the study design did not allow them to account for trends over time [20]. Our results are promising as they indicate clinician behaviors can be improved and sustained in the year following their introduction. Indeed, clinical fidelity-based outcomes (e.g., where the services themselves are monitored directly) may, in fact, be better indicators of quality of care compared to standard facility usage outcomes that most studies use as proxies [26–29]. These positive findings are in line with many other studies from LMICs that used continuous quality improvement strategies to improve maternal and neonatal health services [30–32].

However, though we did find improvements in crude means in the post-intervention period compared to pre-intervention, our evaluation found limited impacts on trends in service utilization (e.g., ANC visits, skilled delivery coverage, PNC follow up, etc.). There are several possible reasons for these results. First, several other studies had similar findings where improvements in point of care quality did not extend into service coverage outcomes [20, 26]. Theoretically, it may be that the quality of care must first improve in order to ‘pull’ more women into care. Thus, a much longer follow up period may be necessary. Moreover, despite a focus on improved data quality within the government health system, data accuracy and precision remains a substantial challenge and may confound results if, for example, more mortality events are truthfully reported and service coverage indicators (e.g., skilled birth attendance) are no longer inflated. On the other hand, many of the change ideas did specifically target coverage (e.g., strategies for tracking women lost to follow up during ANC and improving PNC coverage). While we do believe that quality of care is an important driver of service utilization, it may be that increasing quality of services alone is not enough to encourage mothers into facility-based care, particularly because maternal care seeking and decision making is complex and situated within broader social, cultural, and structural contexts. The evaluation also identified important facility-based indicators that may require particular attention in order to maximize the intervention’s impact. Facilities that had more essential medicines and supplies related to maternal neonatal care, had improved time trends in syphilis testing, skilled delivery, and ANC coverage. Ensuring that facilities have the capacity to provide comprehensive and quality care is of course important, and whenever possible, should be enhanced alongside QI initiatives. Finally, though we did not find significant effects of the amount of QI coaching visits on outcome indicator trends, health workers did report that these were valuable components of the intervention package.

Our study has several strengths, including the attention to validating the quality of facility data, repeated monthly data allowing for a rigorous longitudinal analytic approach, and following facilities for several months both before, during, and after the intervention. Additionally, we have more than 12 months of pre-intervention data, allowing us to account for potential seasonal effects. Of course, attention to data quality may be an intervention in itself and may have impacted the trajectory that was seen before the full implementation of the intervention.

There are also several limitations to consider in the context of this study. First, there are no comparison facilities (due to the difficulty in finding a locations (a) without ongoing potentially confounding interventions and (b) with available high quality data for all outcomes, including the safety bundles), thus limiting our ability to adjust for possible confounders. Second, the limited sample of the pilot study does not allow us sufficient power to detect differences in mortality or other rare events (such as neonatal resuscitation). Third, bundle compliance is contingent on the availability of some supplies (e.g., vitamin K). We cannot account for supply shortages that episodically occur throughout country, which may confound some of our findings and results should thus be interpreted with this limitation in mind; however, given that supply shortages are a typically encountered scenario in LMICs and QI approaches have potential to improve stock management the results are promising. Finally, the improvement in health facility data quality may have resulted increased reporting of mortality events and decreased false inflation of other outcomes (e.g., skilled delivery), thus making it more difficult to find significant impacts. Despite these limitations, the preliminary findings indicate early promise of quality improvement practices on the provision of maternal and newborn health services. Future research should continue to follow the implementation and impacts of QI interventions as they are brought to scale, and track outcomes in maternal and neonatal health domains as well as care quality domains, including patient care perception, clinical service fidelity, and provider perceptions.

## Conclusion

This study points to immediate impacts that QI interventions can have in the facility, but that it may require (1) more follow up time to see impacts on service coverage in communities, (2) continued and sustained QI implementation within facilities. This research demonstrates the feasibility of complex, low-cost, health-worker driven improvement interventions that can be adapted in similar settings around the world. Policy makers and health system workers should carefully consider what our findings mean for scaling QI approaches in Ethiopia and other similar settings.

## Supplementary information

**Additional file 1: Table S.1.** Essential maternal and neonatal medicine and equipment index. **Table S.2.** Maternal and neonatal clinical care bundles.

## Data Availability

Data is available upon reasonable request directed to the senior author HM.

## Abbreviations

QI: Quality improvement
HSTP: Health Sector Transformation Plan
IHI: Institute for Healthcare Improvement
FMoH: Federal Ministry of Health in Ethiopia
SNNPR: Southern Nations, Nationalities, and Peoples’ Region
QITs: Quality improvement teams
HMIS: Health management information system
ANC: Antenatal care visits
PNC: Postnatal care
WHO: World Health Organization
LMIC: Low and middle income countries
MCH: Maternal and child health
