# Supplementary material for: The impacts of quality improvement on maternal and newborn health: preliminary findings from a health system integrated intervention in four Ethiopian regions
Source: BMC Health Serv Res. 2020 Jun 8;20:522. doi: 10.1186/s12913-020-05391-3 (PMC7282234; doi:10.1186/s12913-020-05391-3)
Supplement: Supplementary file 1 — Additional file 1: Table S.1. Essential maternal and neonatal medicine and equipment index. Table S.2. Maternal and neonatal clinical care bundles. [file 12913_2020_5391_MOESM1_ESM.docx]

**Supplementary Material**

**S.1: Essential maternal and neonatal medicine and equipment index**

| Dexamethasone/bethamethasone IV available |
| --- |
| Methyldopa- 1st line available |
| Hydralazine available |
| Nifedipine available |
| Ampicillin (IV) available |
| Amoxicillin (PO) available |
| Gentamycin (IV) available |
| Metronidazole (IV) available |
| Pen G available |
| Ceftriaxone available |
| TTC eye ointment available |
| Vitamin K available |
| MgSO4 available |
| Calcium gluconate available |
| Oxytocin available |
| Misoprostol available |
| Normal saline available |
| Ringers lactate available |
| HIV test kits available |
| Syphilis test kits available |

**S.2: Maternal and neonatal clinical care bundles**

| **Clinical Bundle** | **Components** |
| --- | --- |
| On-Admission | Danger sign assessment  Partograph initiated when cervical dilation at least 4 cm  availability of soap and water/alcohol hand rub, and gloves  Birth companion encouraged to be present during labor and at birth  Mother’s privacy maintained during labor and delivery |
| Before Pushing | Availability of gloves, soap/antiseptic and clean water  Preparation of 10 IU IV/IM Oxytocin in syringe  Availability of two clean, dry, warm towels and suction device  Availability of bag and mask (size 0 and 1)  Helper/Assistant identified and informed for resuscitation |
| Just after Birth (within one hour) | Newborn assessment  Immediate skin to skin and initiate breastfeeding within the 1st hour  Baby weighed and recorded  Administer Vitamin K  Administer tetracycline eye ointment |
